# Encapsulation of Citral by Sodium Carboxymethyl Starch and Sodium Caseinate: Antibacterial Activity Characterization and Evaluation

**DOI:** 10.3390/foods15091492

**Published:** 2026-04-24

**Authors:** Jinfang Hu, Hui Wang, Lufeng Wang, Xuerui Li

**Affiliations:** 1 College of Food Science and Technology, Huazhong Agricultural University, Wuhan 430070, China; 18627256811@163.com; 2 Institute of Agro-Products Processing Science and Technology, Yunnan Academy of Agricultural Sciences, Kunming 650205, China; wh@yaas.org.cn

**Keywords:** sodium carboxymethyl starch, sodium caseinate, citral-loaded microcapsule, antibacterial mechanism, *Staphylococcus aureus*

## Abstract

Citral exhibits favorable broad-spectrum antibacterial activity; however, it is prone to oxidative degradation or structural changes. To improve its stability and practical applicability, citral-loaded microcapsules were prepared using sodium carboxymethyl starch (CMS) and sodium caseinate (CS) via emulsification and freeze-drying. We then investigated the effects of the CMS-to-CS mass ratio on the physicochemical properties and microstructure of the microcapsules, and systematically evaluated the antibacterial activity and underlying mechanisms of the citral-loaded microcapsules against typical foodborne pathogenic bacteria and food-related bacteria. The results showed that when the CMS-to-CS mass ratio was 3:1, the microcapsules prepared exhibited the highest encapsulation efficiency (83.87%). The molecular interactions between citral and the wall materials were confirmed. The citral-loaded microcapsules demonstrated good thermal stability and a compact morphology with dense blocks. Furthermore, treatment with the citral-loaded microcapsules led to the leakage of intracellular contents and compromised the cell membrane integrity of *Staphylococcus aureus*, thereby inhibiting its normal physiological functions, as well as effectively disrupting bacterial aggregation at high concentrations. These findings offer a valuable reference for future studies aimed at improving the stability of citral when used as an antibacterial agent and at enhancing its practical application value.

## 1. Introduction

Citral (CA) is a fat-soluble open-chain monoterpene aldehyde and is composed of two cis-trans isomers, namely geranial and neral [1]. CA has been widely used in the food preservation industry due to its special citrus aroma, distinctive biological activities, and potent antimicrobial and antioxidant properties. For example, it has been demonstrated to possess effective antibacterial activities against *Staphylococcus aureus*, *Escherichia coli*, and *Aspergillus flavus* [2]. However, CA is unstable under various conditions due to its sensitivity to light and heat, which has largely limited its wide application [1]. Biofilms are clusters of microbial cells encased in extracellular polymeric substances. Foodborne pathogens can form biofilms on food-contact surfaces under favorable conditions. Thus, studying the inhibition of biofilms by CA provides insight into its antimicrobial efficacy [3]. Various strategies have been developed to enhance its stability. For instance, microencapsulation, a technology that entraps an active ingredient within a wall material to form particles at the nano-, micro-, or even millimeter-scale, offers an effective approach. Microcapsules can encapsulate bioactive compounds and protect them from humidity, oxygen, light, and other environmental factors. Many antibacterial agents suffer from drawbacks including instability and flavor changes, which can result in decreased efectiveness in products or the generation of off-flavors. Microencapsulation can improve substance stability, control the release of volatile compounds, and mask undesirable odors [4], making it suitable for developing novel food preservatives and freshness-keeping agents [5].

Previous research has documented various encapsulation strategies for CA, including the use of single wall materials such as chitosan or sodium alginate, as well as dual-wall systems such as chitosan-sodium alginate [6]. In another study, a cyclodextrin–metal–organic framework (CD-MOF) was utilized as the wall material to form CDMOF-citral inclusion complex via a vapor diffusion process [7]. CA microcapsules were prepared using dextrin as the encapsulant via spray drying, which demonstrated significant antibacterial effects against *E. coli*, *S. aureus*, and *B. cereus* [8]. To date, relevant research has been primarily focused on exploring different types of wall materials and methods for the microencapsulation of CA, and most studies still remain at the stage of optimizing the preparation processes. However, there have been few reports on the interaction mechanisms between wall materials during microcapsule formation and how they correlate with and regulate the structural and functional properties of the resulting microcapsules.

Sodium carboxymethyl starch (CMS) is an anionic starch ether with well recognized thickening, swelling, and emulsion-stabilizing properties [9]. It can form a three-dimensional network structure by establishing hydrogen bonds with hydrophilic colloids, thereby enhancing system stability [10]. For instance, nanoparticles formed by combining CMS with zein can improve the thermal stability, dispersibility, and antioxidant activity of rutin, while also exhibiting favorable sustained-release characteristics of rutin [11]. In previous studies, sodium caseinate (CS) has often been combined with various materials including γ-cyclodextrin, alginate [12], and Tremella fuciformis polysaccharide [13] for diverse applications. However, the properties of a composite system composed of CMS and CS remain poorly characterized, and there has been no report on the application of this specific combined wall system to the encapsulation of CA in microcapsules to date.

Based on preliminary screening of hydrophilic colloids, this study selected positively charged CS to be composited with CMS for the fabrication of CA-loaded microcapsules via the freeze-drying method, where CA was used as the core material and CMS and CS composed a composite wall material. The study then systematically characterized the physicochemical properties and microstructure of the resultant microcapsules, and evaluated their antibacterial activities against the typical foodborne pathogenic bacteria and food-related bacteria. The findings are expected to offer a valuable reference for future studies aimed at improving the stability of citral when used as an antibacterial agent and at enhancing its practical application value.

## 2. Materials and Methods

### 2.1. Materials and Chemicals

CA was supplied by Shangdong Ke Yuan Biochemical Co., Ltd. (Laizhou, China) CS (BR) and CMS (AR) were obtained from Shangdong Ke Yuan Biochemical Co., Ltd. Petroleum ether was purchased from Changshu Hongsheng Fine Chemical Co., Ltd. (Changshu, China). Luria–Bertani (LB) broth medium and agar powder were obtained from Sangon Biotech Co., Ltd. (Beijing, China) Potato dextrose agar (PDA) culture medium was procured from Beijing Solarbio Science & Technology Co., Ltd. (Beijing, China).

### 2.2. Instruments and Equipment

The following instruments were used in this study: an LGJ-18S freeze dryer (Beijing Songyuan Huaxing Technology Development Co., Ltd., Beijing, China); an Allegra X-30R high-pressure homogenizer (Beckman Coulter, Inc., Indianapolis, IN, USA); an SU3800 scanning electron microscope (Hitachi, Tokyo, Japan); an IS50 Fourier transform infrared (FTIR) spectrometer (Suzhou Ops Plasma Technology Co., Ltd., Suzhou, China); a DSC300 differential scanning calorimeter (NETZSCH-Geratebau GmbH, Selb, Germany); an HVE-50 autoclave (Hirayama Manufacturing Co., Kasukabe-Shi, Japan); an IS-RDV1 constant temperature incubator shaker (Crystal Technology & Industries, Inc., Addison, TX, USA); an LSM 900 laser scanning confocal microscope (Carl Zeiss AG, Oberkochen, Germany); and an SW-CJ-2FD clean bench (Suzhou Antai Air Technology Co., Ltd., Suzhou, China).

### 2.3. Experimental Methods

#### 2.3.1. Preparation of Microcapsules

The microcapsules were prepared with the method described by Zang et al. with minor modifications [14]. Briefly, CMS and CS were separately dissolved in distilled water under high-speed stirring to obtain corresponding stock solutions. The two solutions were then mixed at different CMS-to-CS mass ratios (9:1, 7:1, 5:1, 3:1, 1:1, 1:3, 1:5, 1:7, and 1:9), followed by stirring at 1200 rpm for 15 min to form a homogeneous CMS-CS composite emulsion (Miccra, Buggingen, Germany, MICCRA D-9). The emulsion was subsequently subjected to hydration at 4 °C for 24 h for complete interaction between CMS and CS. After hydration, the mixture was homogenized in a high-speed homogenizer at 10,000 rpm for 15 min (Beckman Coulter, Inc., USA, Allegra X-30R). The homogenized emulsion was then placed in an ice-water bath, and CA was added dropwise at a rate of approximately one drop per second. The mass ratio of the solute in the composite emulsion to the added CA was precisely controlled to form a CA-CMS-CS composite emulsion. This final emulsion was subjected to further homogenization, rapidly frozen in a −80 °C cold trap, and ultimately lyophilized in a vacuum freeze dryer to obtain the CA-loaded microcapsules (Beijing Songyuan Huaxing Technology Development Co., Ltd. LGJ-18S).

#### 2.3.2. Determination of Encapsulation Efficiency

The encapsulation efficiency of the microcapsules was determined with the method described by Zou et al. with minor modifications [15]. Briefly, an accurate mass of 1.000–2.000 g of the prepared microcapsule powder was precisely weighed, and washed with 30 mL of petroleum ether via vortex oscillation for 3 min, followed by filtration. The solid residue on the filter paper was further washed with 10 mL of petroleum ether, which was repeated in triplicate. All filtrates were combined and transferred into a pre-weighed beaker, followed by evaporation of petroleum ether in a 70 °C constant-temperature water bath. When no obvious liquid petroleum ether remained, the beaker was placed in an oven at 70 °C for drying. The mass of the beaker was recorded every 0.5 h until the mass difference between two consecutive weightings was less than 0.1 mg. The surface oil content was subsequently calculated using Equation (1) based on the final mass.

(1) $$\mathrm{Surface}\;\mathrm{oil}\;\mathrm{content}\;\left(m_{1}\right)=\left(\frac{M_{1}-m_{0}}{M}\right)\times 100\%$$

where M_1_ is the mass of the beaker after drying (g), m_0_ is the mass of the empty beaker (g), and M is the mass of the sample (g).

The total essential oil content was determined using the method described by Li et al. with some modifications [16]. An accurately weighed portion of microcapsule powder (1.000–2.000 g) was completely dissolved in 10 mL distilled water at 60 °C. The solution was transferred to a separatory funnel, mixed with 1 mL of ammonia solution, followed by vigorous shaking. Subsequently, 10 mL of absolute ethanol was added and mixed thoroughly, followed by the addition of 25 mL diethyl ether. After shaking and venting, 25 mL of petroleum ether was added. The mixture was left to stand for phase separation. The upper organic layer was collected, and the mixed solvents (petroleum ether and diethyl ether) were evaporated on a water bath at 70 °C. The residue was further dried to a constant weight in an oven at 70 °C. The final mass was precisely recorded, and the total essential oil content was calculated with Equation (2).

(2) $$\mathrm{Total}\;\mathrm{essential}\;\mathrm{oil}\;\mathrm{content}\;\left(m_{2}\right)=\left(\frac{M_{2}-m_{0}}{M}\right)\times 100\%$$

where M_2_ is the mass of the beaker after drying (g), m_0_ is the mass of the empty beaker (g), and M is the mass of the sample (g).

(3) $$\mathrm{Encapsulation}\;\mathrm{efficiency}:\;R=\left(1-\frac{m_{1}}{m_{2}}\right)\times 100\%$$

where M_1_ is the surface oil content (g), and m_2_ is the total essential oil content (g).

#### 2.3.3. Particle Size Determination of CA-Loaded Microcapsules

The particle size was determined according to the method described by Qi et al. [17] with minor modifications. Briefly, 0.1 g of CA-loaded microcapsule samples prepared with different mass ratios was dissolved in 100 mL of distilled water. Then, 0.1 mL of the solution was taken and diluted 100-fold. One milliliter of the diluted solution was used to measure the average particle size using a laser diffraction particle size analyzer (Mastersizer 2000, Malvern Instruments Ltd., Malvern, UK). All measurements were performed in triplicate. The results are presented as the volume-weighted mean diameter D_[4,3]_, and a histogram of the particle size distribution of CA-loaded microcapsules prepared with different mass ratios is plotted accordingly.

#### 2.3.4. Fourier Transform Infrared (FTIR) Spectroscopy Characterization of Microcapsules

The FTIR analysis was carried out with the method described by Li et al. with minor modifications [18]. Briefly, an appropriate amount of microcapsule powder was thoroughly mixed and ground with 150 mg of pre-dried potassium bromide. The mixture was then compressed into a uniform, translucent pellet using a hydraulic press. The FTIR spectra were collected using a Fourier transform infrared spectrometer over the wavenumber range of 400 to 4000 cm^−1^.

#### 2.3.5. Thermal Stability Analysis of Microcapsules

Thermal stability analysis was performed with the method reported by Jiang et al. with minor modifications [19]. A 3–10 mg sample was accurately weighed into an alumina crucible and subjected to characterization using a simultaneous thermal analyzer. The measurement was conducted under a nitrogen atmosphere (flow rate: 50 mL/min) from 30 to 600 °C at a constant heating rate of 10 °C/min.

#### 2.3.6. Morphological Observation

The surface morphology of the microcapsules was observed using a scanning electron microscope (SEM) with minor modifications as described by Karaogul and Ugurtay [20]. Briefly, approximately 10 mg of CA microcapsule powder was evenly dispersed onto a sample stub pre-coated with conductive adhesive. The excess powder was gently removed by air blowing. The stub was then sputter-coated with a thin layer of gold prior to imaging. Observations were performed using an SEM operated at an accelerating voltage of 15 kV.

#### 2.3.7. Determination of Inhibition Zones, MIC, and MBC

For the preparation of culture media, LB broth was prepared by dissolving 2.5 g of LB broth powder in 100 mL of distilled water in a 250 mL reagent bottle. The mixture was stirred to homogeneity and sterilized by autoclaving at 121 °C for 15 min. For LB solid medium, 1.5 g of agar powder was added to the aforementioned mixture prior to autoclaving. Following sterilization, the medium was cooled and poured into sterile disposable Petri dishes. Potato dextrose agar (PDA) solid medium was prepared by dissolving 4.6 g of PDA powder in 100 mL of distilled water, followed by autoclaving at 115 °C for 20 min. The sterilized PDA medium was subsequently cooled and poured into sterile Petri dishes. To prepare bacterial suspensions, a single colony of *S. aureus*, *B. subtilis*, and *L. monocytogenes* was collected from their respective solid media and inoculated into four separate tubes containing LB broth, with the fourth tube serving as a blank control. All tubes were incubated overnight under shaking at a constant temperature. For sample sterilization and preparation, the microcapsule powder was sterilized under UV light for 30 min. A stock solution of the sample was prepared with 5% ethanol for subsequent analyses.

For the inhibition zone test, the bacterial suspension was diluted to 10^5^ CFU/mL and evenly spread onto LB solid medium. Wells with a diameter of 10 mm were punched into the agar using a sterile cork borer. Sterilized sample solutions were then added into the corresponding wells. Following incubation of the plates at a constant temperature for 24 h, the diameter of the inhibition zones was observed, measured, and recorded according to the method reported by Jafari et al. [21].

For minimum inhibitory concentration (MIC) test, the experiment consisted of a sample group, control group 1 (positive control), and control group 2 (solvent control). The bacterial suspensions were adjusted to 10^5^ CFU/mL with LB broth. An initial sample solution at a concentration of 50 mg/mL was prepared and subjected to two-fold serial dilutions in a 96-well plate. Each well was then inoculated with an equal volume of the adjusted bacterial suspension. The plate was incubated at 37 °C for 18 h. Following incubation, the wells were photographed and visually inspected. The lowest concentration of the sample to the first clear well adjacent to turbid wells was recorded as the MIC.

For minimum bactericidal concentration (MBC) test, using the same dilution and incubation protocols as described in the MIC test, the samples were taken from the first turbid well and two preceding wells after incubation. Each sample was spread onto LB agar plates, which were then incubated at 37 °C for 18 h. Following incubation, the number of colonies were counted and recorded. The sample concentration corresponding to the plate showing ≤5 CFU was defined as the MBC.

#### 2.3.8. Determination of Nucleic Acid Concentration

LB broth was prepared by dissolving 2.5 g of LB broth powder in 100 mL of distilled water in a 250 mL reagent bottle. The mixture was stirred to homogeneity and sterilized by autoclaving at 121 °C for 15 min. Two aliquots of the prepared LB broth were transferred into separate test tubes: one was inoculated with a single colony of *S. aureus* picked from a solid medium, while the other remained uninoculated to serve as a blank control. Both tubes were incubated overnight under shaking. The microcapsule powder was sterilized under UV light for 30 min. A stock solution of the sample was subsequently prepared with 5% ethanol for subsequent analyses. After overnight incubation, the bacterial culture was centrifuged at 8000 rpm for 10 min. The bacterial pellet was resuspended in physiological saline and adjusted to a concentration of 10^6^ CFU/mL. The bacterial suspension was treated with the sample solution at 1 × MIC, with an untreated control group set in parallel. According to the method reported by Zhang et al. [22] for optical density measurement, the mixtures were incubated at 37 °C with shaking at 200 rpm for 9 h. At hourly intervals, 0.5 mL aliquots of the culture were sampled, centrifuged at 6000 rpm for 10 min, and the absorbance of the supernatant was measured at 260 nm.

#### 2.3.9. Analysis of Bacterial Biofilm Structure

*S. aureus* suspension was diluted to 10^6^ CFU/mL using LB broth. The diluted suspension was transferred to confocal dishes and incubated at 37 °C for 72 h to facilitate biofilm formation. Microcapsule solutions at concentrations of 1/2 × MIC, 1 × MIC, 2 × MIC, and 4 × MIC, as well as the required staining solutions, were prepared in advance. Following the incubation period, the formed biofilms were gently washed twice with sterile phosphate-buffered saline. The prepared sample solutions were then added to the corresponding confocal dishes, while the control group was treated with LB broth containing 5% ethanol. After 24 h incubation with the samples, the biofilms were washed again twice with sterile PBS. Subsequently, the biofilms were stained by applying SYTO 9 and propidium iodide onto their surface, followed by incubation at 37 °C in the dark for 15 min. After staining, excess dye was removed by washing with PBS. The biofilms were finally observed and imaged using excitation wavelengths of 488 nm and 561 nm [23].

#### 2.3.10. Scanning Electron Microscopy Observation

The bacterial suspension of *S. aureus* was treated with the microcapsule sample solution at a final concentration of 1 × MIC, while the control group was treated with 5% ethanol solution. After incubation at 37 °C for 18 h, the bacterial cells were harvested and fixed overnight at 4 °C with 2% glutaraldehyde. The samples were then rinsed three times with PBS buffer, followed by gradient dehydration in a graded ethanol series (30%, 50%, 70%, 90%, and 100%) for 15 min at each concentration. Subsequently, the samples were dried using a critical point dryer, sputter-coated with gold, and observed under SEM to characterize the morphological alterations of the bacterial cells [24].

### 2.4. Statictical Analysis

Data processing and plotting were performed using Origin 2024 software (OriginLab Corporation, Northampton, MA, USA). Data visualization was performed with GraphPad Prism 8.0 (GraphPad Software, Boston, MA, USA). SPSS 19.0 software was used to perform one-way analysis to identify significant differences among treaments. A least significant difference test was used to determine significant differences in the means at *p* < 0.05.

## 3. Results and Discussion

### 3.1. Citral-Loaded Microcapsule Characterization

#### 3.1.1. Microcapsule Encapsulation Efficiency for CA

**Figure 1:**
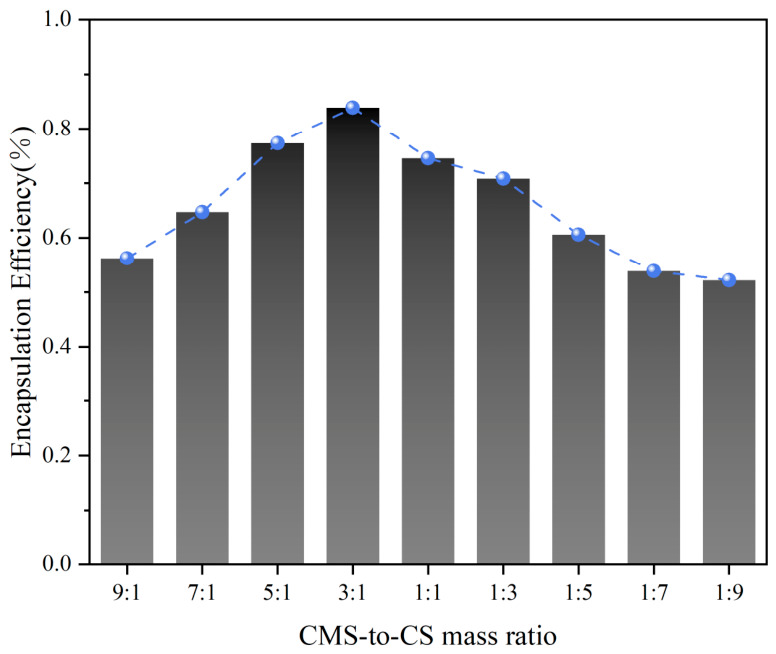
CA encapsulation efficiency of microcapsules with different CMS-to-CS mass ratios.

As known in Figure 1, in the present study, we blended CMS and CS at various mass ratios (9:1, 7:1, 5:1, 3:1, 1:1, 1:3, 1:5, 1:7, and 1:9), which achieved an encapsulation efficiency of 56.25%, 64.71%, 77.36%, 83.87%, 74.55%, 70.83%, 60.61%, 53.85%, and 52.17% for CA, respectively. The encapsulation efficiency reached the maximum (83.87%) at the CMS-to-CS mass ratio of 3:1, and gradually decreased thereafter. This decrease might be ascribed to the fact that an excessive concentration of emulsifier with increasing CS ratio may elevate the emulsion viscosity, restrict molecular mobility, and thereby impede the efficient incorporation of CA into the CMS-CS composite matrix. This is consistent with the previous research findings. A combination of CMS with hydrophilic colloids can enhance the stability of emulsion systems. CS is a positively charged hydrophilic colloid and is expected to improve the system stability upon complexation with CMS. It should be noted that the mass ratio of the two components has a profound influence on the functional performance of the composite matrix [10].

#### 3.1.2. Particle Size Analysis of CA-Loaded Microcapsules

**Figure 2:**
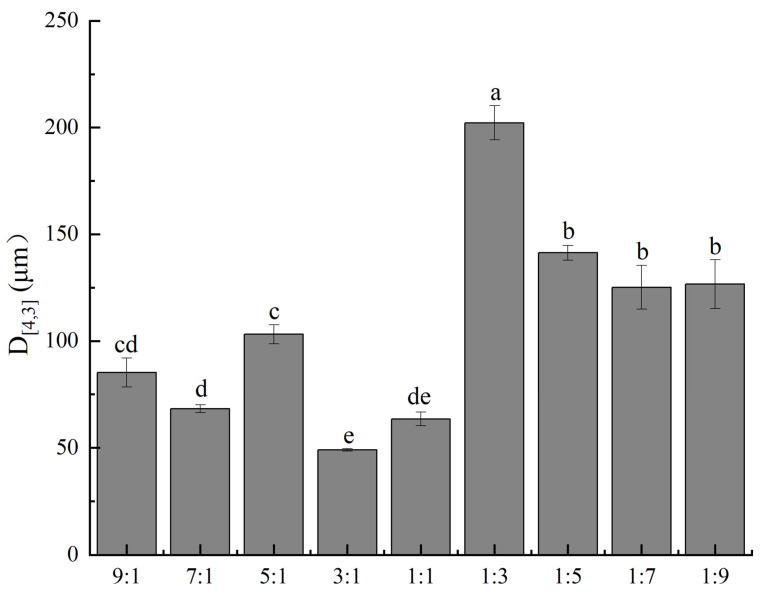
Volume-weighted average particle size of CA-loaded microcapsules with different wall material qualities. Vertical bars indicate the standard errors of three replications. Different lowercase letters at same storage tiome points are significantly different.

Particle size directly influences the encapsulation efficiency, release behavior, and stability of CA-loaded microcapsules, and is therefore a key parameter for evaluating their physical properties. In this study, the particle size distribution of CA-loaded microcapsules prepared with different mass ratios was determined using a laser diffraction particle size analyzer, aiming to assess their physical performance and provide a structural basis for subsequent application in preservation systems. The results are shown in Figure 2. Significant differences in particle size were observed among the CA-loaded microcapsules prepared with different mass ratios. Groups with a higher proportion of CS exhibited markedly larger particle sizes than those with a higher proportion of CMS. This is primarily because CMS is a negatively charged anionic polysaccharide, whereas CS can carry different charges depending on pH conditions. When the CS content is high, the excess positive charge in the composite system weakens the electrostatic repulsion between molecules, making molecular chains more prone to aggregation and thus forming larger particles. In addition, an excessive amount of CS prevents the formation of a stable three-dimensional network structure with CMS, resulting in a loose interfacial film that cannot effectively resist droplet coalescence. Furthermore, hydrophobic interactions and hydrogen bonding among protein molecular chains promote molecular aggregation, which directly leads to an increase in the particle size of CA-loaded microcapsules. The smallest particle size (49.045 um) was observed at a CMS-to-CS mass ratio of 3:1. This indicates that at this ratio, the electrostatic complexation between CMS and CS is most effective, producing the most compact and uniform interfacial film structure, which can efficiently encapsulate citral into fine particles and resist coalescence under drying conditions. From the perspective of drug release, a smaller particle size of CA-loaded microcapsules corresponds to a larger specific surface area, which leads to a faster release rate of the core material, whereas a larger particle size prolongs the diffusion path of the core material. These findings further confirm that the CA-loaded microcapsules prepared at the 3:1 mass ratio exhibit the best encapsulation performance and citral release behavior, making it the optimal ratio.

#### 3.1.3. FTIR Spectra of Microcapsules

**Figure 3:**
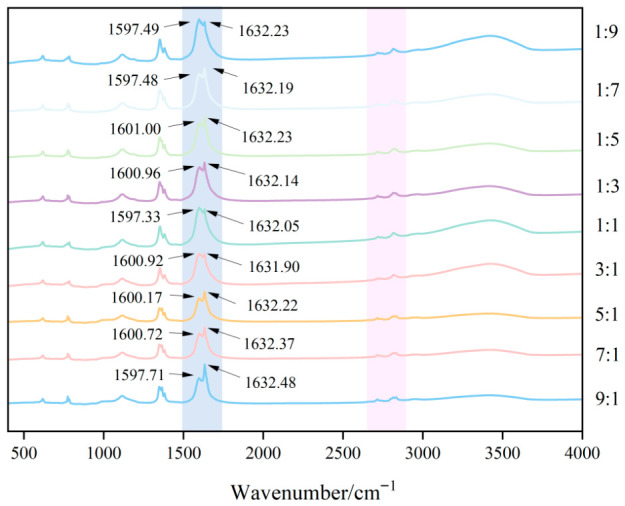
FTIR spectra of CA-loaded microcapsules with different CMS-to-CS mass ratios.

FTIR spectroscopy was utilized to characterize the variations in functional groups and intermolecular interactions within the biopolymer matrix (Figure 3). The characteristic FTIR absorption peaks of CMS included the O–H stretching vibration (~3400 cm^−1^), C–H stretching vibrations of methyl and methylene groups (~2920 cm^−1^ and ~2850 cm^−1^), and C=O stretching vibration of the carboxyl group (~1610–1630 cm^−1^) [25]. CS showed characteristic peaks corresponding to the overlapping N–H and O–H stretching vibration (~3400 cm^−1^), C–H stretching vibrations (~2920 cm^−1^ and ~2850 cm^−1^), and the amide I band derived from the C=O stretching vibration of peptide bonds (~1630–1650 cm^−1^) [26]. These results indicated that CMS and CS have highly similar peak positions. For CA, the characteristic absorption peaks are ascribed to the C=C stretching vibration (~1640–1680 cm^−1^) and the aldehyde C–H stretching vibrations (~2720–2740 cm^−1^ and ~2820 cm^−1^) [27]. The FTIR spectra of the CA-CMS-CS microcapsules prepared at different mass ratios are presented in Figure 3. The spectra displayed characteristic peaks corresponding to the C=O stretching vibration at approximately 1600 cm^−1^ and the overlapping O–H/N–H stretching vibration around 3400 cm^−1^, which represent the key fingerprint features of the CMS and CS wall materials. Furthermore, the characteristic peaks attributed to CA were also observed, including the C=C stretching vibration near 1630 cm^−1^ and the distinctive aldehyde C–H stretching vibrations at approximately 2717 cm^−1^ and 2815 cm^−1^. This is consistent with the previous research findings. While the overall spectral profiles generally remained consistent, a special trend was noted for the peak intensity at 1630 cm^−1^ (assigned to the C=C vibration of CA), which first decreased and then increased with increasing CS ratio, and reached the minimum at the CMS-to-CS mass ratio of 3:1. This attenuation trend of the CA characteristic peak is most likely ascribed to its effective encapsulation within the composite wall matrix. This phenomenon is in good agreement with the results for encapsulation efficiency, where the maximum encapsulation efficiency (83.87%) was obtained at the 3:1 CMS-to-CS mass ratio. The subsequent increase in peak intensity at higher CS ratios corresponds well with the downward trend of encapsulation efficiency, implying weakened shielding and less effective entrapment of CA within the microcapsules.

#### 3.1.4. Citral-Loaded Microcapsules Thermal Stability Analysis

**Figure 4:**
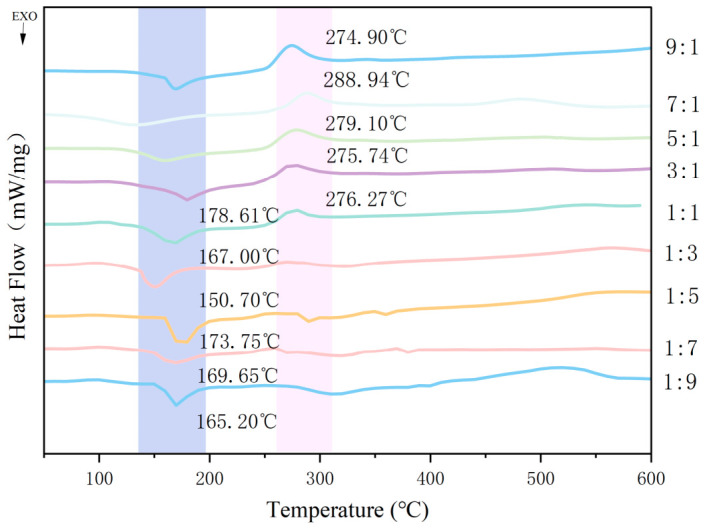
DSC curves of CA-loaded microcapsules with different CMS-to-CS mass ratios.

The DSC curves of CA-loaded microcapsules prepared at different CMS-to-CS mass ratios are displayed in Figure 4. For samples with CMS-to-CS ratios of 9:1, 7:1, 5:1, 3:1, and 1:1, a single endothermic peak was observed during the heating process at the temperature of 274.90 °C, 288.94 °C, 279.10 °C, 275.74 °C, and 276.27 °C, respectively. These endothermic characteristics probably correspond to the melting temperature of the microcapsule matrix. In contrast, samples with CMS-to-CS mass ratios of 1:9, 1:7, 1:5, 1:3, 1:1, and 3:1 exhibited a single exothermic peak during the heating process at the temperature of 165.20 °C, 169.65 °C, 173.75 °C, 150.70 °C, 167.61 °C, and 178.61 °C, respectively. This phenomenon is probably associated with the oxidation of CA released from the microcapsules with increasing temperature [28]. These results indicated that the CA-loaded microcapsules could remain relatively stable at temperatures below 150 °C. This provides guidance for the actual application scenarios of this microcapsule. Among all samples, the sample with a CMS-to-CS mass ratio of 3:1 exhibited the highest exothermic peak temperature (178.61 °C), implying that it possesses the most superior thermal stability.

#### 3.1.5. Morphology of Microcapsules

**Figure 5:**
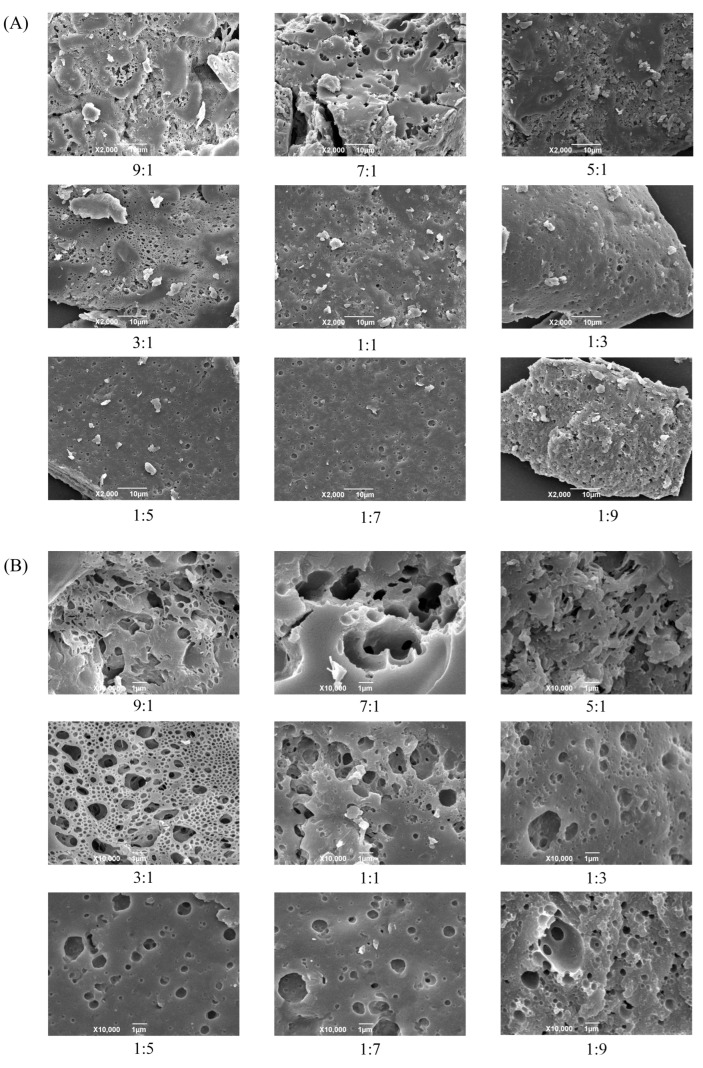
SEM Images of CA-loaded microcapsules with different CMS-to-CS mass ratios ((**A**): ×2000, (**B**): ×10,000).

Figure 5 displays the SEM images of CA-loaded microcapsules with different CMS-to-CS mass ratios. The CA-loaded microcapsules showed irregular blocks of varying sizes on the surface, while pristine CMS and CS exhibited a uniform spherical morphology, indicating that significant intermolecular interactions occurred between CA and the CMS-CS composite matrix during the encapsulation process. It can be preliminarily speculated that intermolecular interactions among the three components may occur during the freeze-drying procedure, which modifies the morphology of CMS and CS and generates a new phase, ultimately resulting in a distinctive microcapsule structure. Furthermore, at identical magnification, the surfaces of microcapsules prepared at all CMS-to-CS mass ratios displayed pores and channels with diverse diameters. With increasing ratio of CS, the number and size of these surface pores gradually decreased. The microcapsules with CMS-to-CS mass ratios of 9:1, 7:1, and 5:1 exhibited fairly rough surfaces with complex architectures and heterogeneous pore size distributions, which could be attributed to the excessively high CMS ratios and inadequate emulsifier (CS) dosage. In contrast, the microcapsules with a CMS-to-CS mass ratio of 3:1 possessed more uniformly distributed pores, as well as a smaller average diameter and more orderly pore size distribution, which is in good agreement with the DSC analysis results.

### 3.2. Antibacterial Mechanism Analysis of Microcapsules

#### 3.2.1. Analysis of Inhibition Zone, MIC, and MBC

Measurement of inhibition zones revealed that the CA-loaded microcapsules displayed significant antibacterial activity against *S. aureus*, *B. subtilis*, and *L. monocytogenes*, particularly against *B. subtilis*. This observation validated the efficient release of high concentrations of bioactive compounds from the microcapsules during incubation. MIC, the lowest concentration capable of inhibiting 99.9% of microbial growth, can reflect the antimicrobial potency of an agent, with a lower value signifying a stronger inhibitory efficacy [21]. As known in Table 1, the MIC and MBC assay results revealed that the MIC of CA-loaded microcapsules was 6.25, 3.13, and 6.25 mg/mL against *S. aureus*, *B. subtilis*, and *L. monocytogenes*, with corresponding MBC values of 12.5, 6.25, and 25 mg/mL, respectively. Notably, the MBC for the first two bacteria was exactly twice their MIC. Numerous studies have demonstrated that CA exhibits inhibitory activities against plant pathogens, foodborne pathogens, wood-decay fungi, and molds [29], making it a promising candidate for antimicrobial preservation in food applications. Nevertheless, CA possesses unsaturated bonds such as C=C and C=O moieties, which endow it with high instability and volatility. Encapsulation of CA within microcapsules can improve its stability, achieve controlled release of its antimicrobial constituents, and therefore enhance its antibacterial efficacy.

**Table 1:** Minimum inhibitory concentration and minimum bactericidal concentration of CA-loaded microcapsules against typical foodborne pathogenic bacteria and food-related bacteria.

	*S. aureus*	*B. subtilis*	*L. monocytogenes*
MIC	6.25 mg/mL	3.13 mg/mL	6.25 mg/mL
MBC	12.5 mg/mL	6.25 mg/mL	25 mg/mL

#### 3.2.2. Effect of Microcapsules on Bacterial Nucleic Acid Concentration

**Figure 6:**
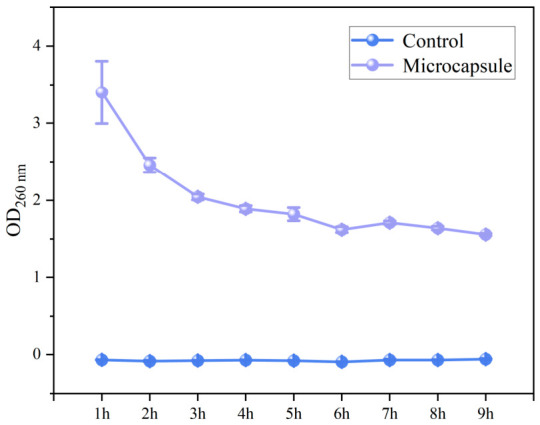
Changes in nucleic acid macromolecules in the supernatant of *Staphylococcus aureus* culture medium.

The results of OD_260_ measurement are presented in Figure 6. As the incubation time of *S*. *aureus* increased, the nucleic acid concentration in the control group exhibited no significant variation, which is indicative of normal bacterial growth; in contrast, that in the supernatant of the *S*. *aureus* suspension treated with CA-loaded microcapsules continuously decreased over time. 1× MIC was sufficient to completely inhibit bacterial growth. At the initial stage of treatment, transient cell membrane damage and nucleic acid leakage probably occurred, resulting in a relatively high initial OD_260_ value. Subsequently, the bacteria showed progressive death and autolysis due to metabolic inhibition. The nucleases released from the lysed cells might degrade the nucleic acids, contributing to the observed continuous decrease in OD_260_ value in later incubation. The destruction of DNA can impede gene expression, ultimately causing bacterial death [22]. The progressively decreasing OD_260_ value suggested DNA fragmentation, demonstrating that the CA-loaded microcapsule treatment not only inhibits *S. aureus* growth but also induces its cell death and nucleic acid degradation via the sustained release of CA, this is consistent with the previous research findings [22].

#### 3.2.3. Effect of Microcapsules on Bacterial Biofilm Permeability

**Figure 7:**
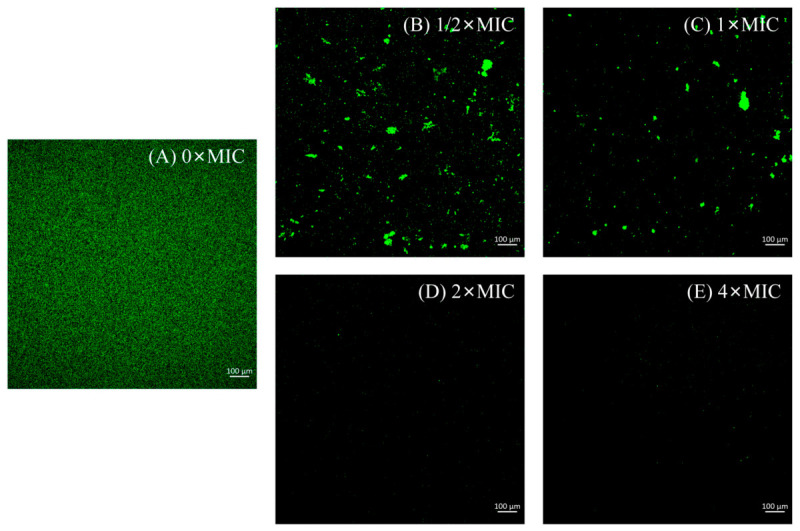
Inhibitory effects of CA-loaded microcapsules at different folds of MIC on *Staphylococcus aureus* biofilms.

Figure 7 shows the biofilms formed by *S*. *aureus* in both the control group (0× MIC) and groups treated with microcapsule solutions at 1/2× MIC, 1× MIC, 2× MIC, and 4× MIC. In CLSM analysis, a reduction or shrinkage of fluorescent regions signifies a decrease in the bacterial population within the biofilm [30]. In the 0× MIC group, the *S. aureus* biofilm displayed a dense and intact three-dimensional architecture, where all cells existed as viable single units with no visible aggregates. In the 1/2× MIC and 1× MIC groups, the three-dimensional architecture of the biofilm was completely disrupted, accompanied by a significantly diminishing count of single cells. Concurrently, a small number of green fluorescent aggregates emerged, implying that the bacterial cells initiated a collective resistance mechanism in response to the hostile environment induced by the CA-loaded microcapsules, which probably involves enhancement of cell-to-cell adhesion to counteract the penetration and antibacterial action of CA. In the 2× MIC and 4× MIC groups, only a minimal number of single cells were observable, demonstrating that the CA-loaded microcapsules can not only kill individual bacterial cells but also effectively dismantle the aggregated structures formed by the bacteria. Overall, the bactericidal efficacy of the CA-loaded microcapsules is concentration-dependent: at sub-inhibitory concentrations, they induce bacterial aggregation as a defensive response, whereas at high concentrations, they completely dismantle bacterial aggregates and eradicate the residual bacteria. These findings reveal the antimicrobial mechanism of the CA-loaded microcapsules from the perspectives of both cellular morphology and structural integrity.

#### 3.2.4. Effect of Microcapsules on Bacterial Morphology

**Figure 8:**
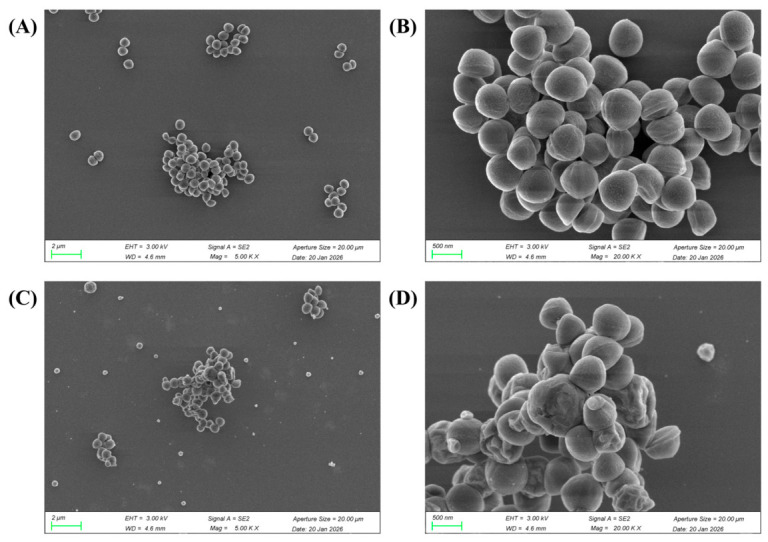
Scanning electron micrographs of *Staphylococcus aureus*. (**A**,**B**): Control group, *S. aureus* cells untreated with CA-loaded microcapsules. (**C**,**D**): *S. aureus* cells treated with 1× MIC.

SEM observations revealed distinct morphological alterations in *S*. *aureus* cells after treatment with CA-loaded microcapsules. As known in Figure 8, the untreated cells (control group) were plump with comparatively smooth surfaces, exhibiting typical, intact spherical morphological characteristics of staphylococcal cocci, where no cell lysis or leakage of intracellular contents was detected. In contrast, co-culturing with CA-loaded microcapsules at 1× MIC resulted in distinct morphological changes in *S. aureus*. The bacterial surfaces became rough and uneven, exhibiting widespread shrinkage with an irregular spherical shape. These observations suggested that CA probably interferes with the synthesis or cross-linking of peptidoglycan within the bacterial cell wall [29]. Furthermore, pronounced concavities were visible on the surface of many cells. In severe cases, excessive indentation can lead to cell rupture and conspicuous leakage of intracellular contents. CA is a hydrophobic terpenoid aldehyde, and may be integrated into the lipid bilayer of the bacterial cytoplasmic membrane, thereby elevating its permeability. Such disruption will give rise to an imbalance in transmembrane ion gradients, ultimately compromising the membrane integrity. Therefore, it can be inferred that CA inhibits the normal growth of *S*. *aureus* by impeding cell wall biosynthesis and compromising the cell membrane integrity, this is consistent with the previous research findings [29].

## 4. Conclusions

This study investigated the physicochemical properties, thermal stability, and antibacterial efficacy of CA-loaded microcapsules fabricated with a composite wall system consisting of CMS and CS. A CMS-to-CS mass ratio of 3:1 was identified as the optimal ratio, which resulted in the most uniform distribution of pore size and number on the microcapsule surface, the lowest intensity of the characteristic peak of CA, and thereby an encapsulation efficiency exceeding 80%, as well as excellent thermal stability of the microcapsule at temperatures up to 150 °C. Moreover, the CA-loaded microcapsules exhibited potent antibacterial activities against typical foodborne pathogenic bacteria and food-related bacteria, specifically *S. aureus*, *B. subtilis*, and *L. monocytogenes*. Microplate assays indicated that the CA-loaded microcapsule treatment induced cell death in *S. aureus* and promoted nucleic acid degradation through the sustained release of CA. The CA-loaded microcapsules could not only eradicate planktonic bacterial cells but also effectively disrupt the aggregated biofilm architecture, probably through cleavage of DNA and impairment of cell membrane integrity. These modifications alter the cellular morphology and structural integrity of *S. aureus*, thereby interfering with its intrinsic physiological functions and ultimately culminating in cell death. These findings offer a valuable reference for future studies aimed at improving the stability of citral when used as an antibacterial agent and at enhancing its practical application value.

## Data Availability

The original contributions presented in this study are included in the article. Further inquiries can be directed to the corresponding authors.
